# Art and Aging: Use of Social Media for Geriatric Education

**DOI:** 10.1093/geroni/igab046.2810

**Published:** 2021-12-17

**Authors:** Gunjan Manocha, Casey Morton, Nicole Derenne, Heidi Bau, Donald Jurivich

**Affiliations:** University of North Dakota, Grand Forks, North Dakota, United States

## Abstract

Social media as an educational tool for health care learning has untapped potential. Benefits of social media include peer-to-peer engagement, active learning and interprofessional training. Here we explored social media platforms as a vehicle to deliver short, pithy clinical pearls from evidence-based, peer-reviewed manuscripts. Key points from recent medical publications are paired with pre-existing artwork to provide visual reinforcement of the clinical pearl. Dubbed “Art and Aging”, the clinical pearl and artwork combination is posted on different social media platforms such as Instagram, Twitter and Facebook, thus allowing for an expansive audience. Different hashtags and tags are used to increase followers and engagement on each platform. Over a 9 months period learner engagement increased by 150% and includes a diverse learner profile. These curated social media platforms show considerable promise for disseminating Geriatrics best practices. As yet, we do not know subject matter retention or whether it changes clinical practices - both questions which are future research objectives.

